# PfIRR Interacts with HrIGF-I and Activates the MAP-kinase and PI3-kinase Signaling Pathways to Regulate Glycogen Metabolism in *Pinctada fucata*

**DOI:** 10.1038/srep22063

**Published:** 2016-02-25

**Authors:** Yu Shi, Mao-xian He

**Affiliations:** 1 CAS Key Laboratory of Tropical Marine Bio-resources and Ecology, Guangdong Provincial Key Laboratory of Applied Marine Biology, South China Sea Institute of Oceanology, Chinese Academy of Sciences, 164 West Xingang Road, Guangzhou 510301, China

## Abstract

The insulin-induced mitogen-activated protein kinase (MAPK) and phosphatidylinositol 3-kinase (PI3K) pathways are major intracellular signaling modules and conserved among eukaryotes that are known to regulate diverse cellular processes. However, they have not been investigated in the mollusk species *Pinctada fucata*. Here, we demonstrate that insulin-related peptide receptor of *P. fucata* (pfIRR) interacts with human recombinant insulin-like growth factor I (hrIGF-I), and stimulates the MAPK and PI3K signaling pathways in *P. fucata* oocytes. We also show that inhibition of pfIRR by the inhibitor PQ401 significantly attenuates the basal and hrIGF-I-induced phosphorylation of MAPK and PI3K/Akt at amino acid residues threonine 308 and serine 473. Furthermore, our experiments show that there is cross-talk between the MAPK and PI3K/Akt pathways, in which MAPK kinase positively regulates the PI3K pathway, and PI3K positively regulates the MAPK cascade. Intramuscular injection of hrIGF-I stimulates the PI3K and MAPK pathways to increase the expression of pfirr, protein phosphatase 1, glucokinase, and the phosphorylation of glycogen synthase, decreases the mRNA expression of glycogen synthase kinase-3 beta, decreases glucose levels in hemocytes, and increases glycogen levels in digestive glands. These results suggest that the MAPK and PI3K pathways in *P. fucata* transmit the hrIGF-I signal to regulate glycogen metabolism.

The insulin signaling pathway, one of the most widely distributed pathways among invertebrate species, is conserved among eukaryotes, including mammals, *Caenorhabditis elegans*, and *Drosophila*^1^,^2^,^3^, and it has been shown to be involved in the regulation of many important physiological responses, including glucose levels^1^, animal growth^4^, development^5^, metabolism^6^, reproduction, and cell life span^3^,^7^.

The insulin signal is transmitted via the insulin receptor (IR). Vertebrates possess more than one homologous IR family member, including the IR, the type-I insulin-like growth factor receptor (IGF-IR), and the insulin receptor-related receptor (IRR)^8^,^9^,^10^, whereas invertebrates possess only a single one — the insulin-related peptide receptor (IRR)^1^. Activation of the IR is linked to two major intracellular signaling pathways, the Ras/Raf/mitogen activated protein kinase (MAPK) kinase (MEK)/extracellular signal-regulated kinase 1 and 2 (ERK1/2) pathway and the phosphatidylinositol 3-kinase (PI3K)/phosphoinositide-dependent kinase 1 (PDK1)/Akt signaling pathway^11^,^12^. Although the MEK/ERK and PI3-kinase/Akt signaling pathways have been studied extensively, the cross-talk between these two pathways is not well understood. In this context, experimental data and computer simulations demonstrate that cross-talk is context-dependent, and that both pathways can activate or inhibit each other^13^.

Numerous insulin signaling pathways in invertebrates and vertebrates have been found to be relatively well conserved, including the ligand, its receptors, and the final effectors^14^,^15^,^16^. In invertebrates, numerous orthologs of the insulin pathway effectors have been shown to be highly conserved during evolution in ecdysozoan species such as *Drosophila melanogaster* and *C. elegans*^16^,^17^,^18^.

In mollusks, insulin-like peptides (ILPs) have been found in *Lymnaea stagnalis* (seven ILPs)^19^,^20^,^21^, *Aplysia californica* (one ILP)^22^, *Anodonta cygnea* (six ILPs)^23^, *Lottia gigantea* (four ILPs)^24^, and *Crassostrea gigas* (one ILP)^25^; IRRs, which share the common characteristic of a typical tyrosine kinase (TK) domain, have been identified only in a few mollusks^6^,^26^,^27^, and they are highly conserved in vertebrates. The affinity of the IRR of the mussel *A. cygnea* for recombinant piscine IGF-I and porcine insulin has been studied. The results indicate that this receptor shares similar binding properties with vertebrates IRRs^28^. There have been few studies of the other elements of the insulin pathway in mollusks. Ras has been identified only in *Mytilus trossolus*^29^ and *A. californica*^30^. Recently, in *C. gigas*, CgRas, CgPten, and CgP70S6K were found based on their sequence conservation^31^. This evidence suggests that the conservation of the insulin signaling system may extend throughout bivalve species.

Insulin can stimulate the PI3K and MAPK pathways to increase glucose transport and glycogen and lipid synthesis, diminish gluconeogenesis, inhibit glycogenolysis and lipolysis, and regulate the expression of specific genes^32^. Glycogen synthesis is regulated by the enzyme glycogen synthase (GS)^33^, whose activity is regulated by GS kinase 3 (GSK3)^34^. Upon phosphorylation by Akt, GSK3 (both the alpha and beta isoforms) catalytic activity is turned off, resulting in the activation of pathways that are normally repressed by GSK3^7^. Insulin activates GS by inhibiting GSK3 and activating protein phosphatase 1 (PP1)^35^. Previous studies have reported that exogenous insulin stimulates glycogen accumulation via the PI3K/AKT pathway in invertebrates such as *Rhipicephalus (Boophilus) microplus*^36^, the white shrimp *Penaeus vannamei*^37^, and the lobster *Panulirus argus*^38^.

The pearl oyster, *Pinctada fucata*, a marine bivalve mollusk that is mainly cultivated in China and Japan, has very high economic value in terms of pearl production, and it is also an important research topic regarding biomineralization, growth, genetic variation, and resistance to disease. In our previous study, we characterized the insulin-related peptide receptor of *P. fucata* (*pfirr*) gene, which is involved in regulating developmental processes in *P. fucata*^39^, and demonstrated that its structure and function are conserved. However, the mechanism of regulation and the signaling pathway mediated by pfIRR have not been studied in *P. fucata*. Moreover, the MAPK and PI3K signaling pathways have not been studied in *P. fucata*. Additionally, it is unknown whether pfIRR can regulate these signaling pathways, and whether these two pathways interact with each other. Furthermore, it is not known whether the insulin/IGF-I responsive machinery in *P. fucata* is correlated with carbohydrate/glycogen metabolism via these signaling pathways.

To investigate the insulin signaling pathway in *P. fucata*, we first tested the interaction between human recombinant IGF-I (hrIGF-I) and pfIRR, and the effect of hrIGF-I-mediated pfIRR stimulation in activating the MAPK and PI3K signaling cascades in *P. fucata* oocytes. Second, we examined the effect of the pfIRR inhibitor PQ401 on hrIGF-I-mediated Akt/protein kinase B (PKB) and MAPK phosphorylation. Third, we measured the effects of the MEK inhibitor PD98059 and the PI3K inhibitor wortmannin on the activation of the hrIGF-I-induced PI3K and MAPK signaling pathways downstream of pfIRR in *P. fucata* oocytes. Fourth, we analyzed the effects of hrIGF-I on glycogen content and glucose levels, the phosphorylation of Akt/PKB at amino acid residues Thr308 (T308) and Ser473 (S473), as well as p44/42 MAPK, and the expression of genes following the intramuscular (i.m.) injection of hrIGF-I.

## Results

### Characterization of an anti-IRR polyclonal antibody

**Figure 1:**
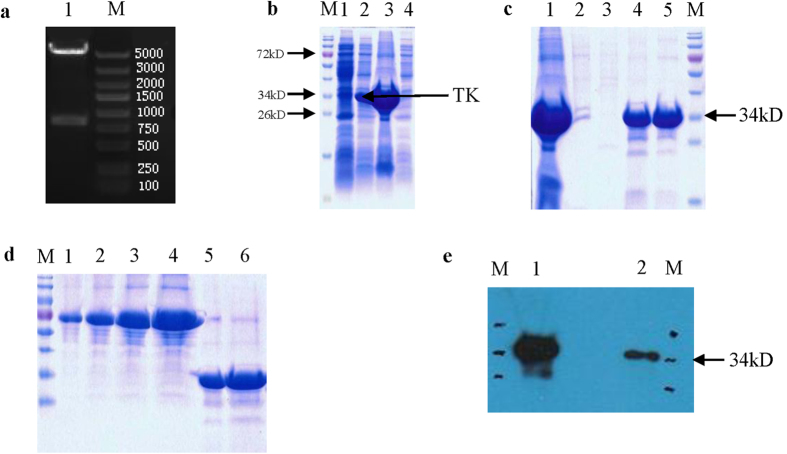
Production of the TK domain of the IRR of *P. fucata* polyclonal antibody. (**a**) pET28a plasmid was digested with two restriction enzymes (BamHI and XhoI) to check the successful cloning of the TK-encoding domain into a bacterial expression system. Lane 1, pET28a-TK digested by BamHI and XhoI; lane M, DNA Marker. (**b**) 15% SDS-PAGE analysis of recombinant TK after IPTG induction. Lane 1, total protein from uninduced *E. coli* harboring pET28a-TK; lane 2, total protein from induced *E. coli* harboring pET28a-TK (28 °C, 4 h after 0.5 mM IPTG induction); lane 3: the insoluble fraction after ultrasonication precipitation; lane 4, the soluble fraction after ultrasonication. (**c**) Purification of recombinant TK. Lane 1, the supernatant of the ultrasonication precipitate after solubilization in 8 M urea (used for TK purification); lane 2, the flow through; lane 3, the elution of NTAU-10, Lane 4: the elution of NTAU-50, Lane 5: the elution of NTAU-200. (**d**) Quantitative analysis of recombinant TK. Lane 1, BSA standard (1 μg); lane 2, BSA standard (2 μg); lane 3, BSA standard (4 μg); lane 4, BSA standard (8 μg); lane 5, recombinant TK (2 μl); lane 6, recombinant TK (4 μl). (**e**) Evaluation of the anti-TK polyclonal antibody by western blotting. Lane M, protein marker; lanes 1 and 2, purified recombinant TK analyzed by western blotting with 1:5,000 and 1:20,000 dilutions, respectively, of the purified antibody.

After double-digestion with BamHI and XhoI, the 837-bp cDNA fragment encoding the TK domain of IRR was amplified and cloned into the BamHI/XhoI sites of the pET28a expression vector, which results in the fusion of a histidine tag to the TK domain. Then, the TK domain was expressed in *Escherichia coli* and purified (Fig. 1a). Sodium dodecyl sulfate-polyacrylamide gel electrophoresis (SDS-PAGE) analysis revealed that the molecular weight of the TK domain was approximately 34 kDa (Fig. 1b). Following induction with isopropyl β-D-1-thiogalactopyranoside (IPTG), the TK domain was detected almost exclusively in inclusion bodies. (Fig. 1b). The denatured TK domain had better affinity for a Ni^2+^-NTA column, and a higher concentration of the TK domain was eluted with a 50–200 mM imidazole gradient (Fig. 1c). Finally, 1.3 mg/ml of purified TK domain was obtained (Fig. 1d).

Polyclonal antibodies were generated in rabbits using the purified TK domain. The antisera were purified using a protein A affinity column. Antigen titers were detected by an enzyme-linked immunosorbent assay (ELISA) with 1:1,250, 2,500, 5,000, 10,000, 20,000, 40,000, and 80,000 dilutions of the antisera (Table 1). The concentration of the polyclonal antibody serum was 6.76 mg/ml. Western blot analyses were conducted with the purified TK domain. A single band with a molecular weight of approximately 34 kDa was detected (Fig. 1e). These results demonstrate that the antibody specifically recognizes the TK domain of pfIRR.

### hrIGF-I interacts with pfIRR in *P. fucata* oocytes

**Figure 2:**
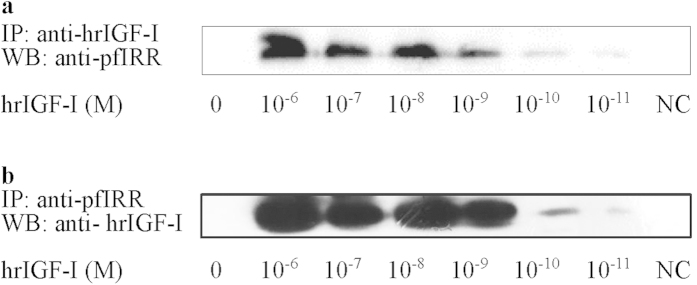
Co-IP analysis of the interaction between hrIGF-I and pfIRR after treatment with various concentrations of hrIGF-I. (**a**) The samples were co-immunoprecipitated using anti-hrIGF-I antibodies prior to analysis by SDS-PAGE (10%). Proteins were detected by western immunoblotting with an anti-pfIRR antibody. The negative control (NC) was rabbit IgG. (**b**) The samples were co-immunoprecipitated using anti-pfIRR antibodies prior to analysis by SDS-PAGE (10%). Proteins were detected by western blotting with an anti-hrIGF-I antibody. The NC was rabbit IgG.

To verify the interaction between hrIGF-I and pfIRR, an *in vitro* co-immunoprecipitation (Co-IP) analysis using *P. fucata* oocytes was employed. The results showed that pfIRR was co-immunoprecipitated by hrIGF-I (Fig. 2a), while hrIGF-I was co-immunoprecipitated by pfIRR (Fig. 2b). The interaction between hrIGF-I and pfIRR was dose-dependent, and it increased with increasing hrIGF-I concentrations (10^−11^–10^−6^ M) when using anti-hrIGF-I antibodies for the Co-IP (Fig. 2a). A slight dose-dependent increase was observed for hrIGF-I concentrations ranging from 10^−9^ to 10^−6^ M, and there was a dose-dependent increase when using anti-pfIRR antibodies (from 10^−11^ to 10^−9^ M) for the Co-IP (Fig. 2b).

### IGF stimulation activates the MAPK and PI3K signaling pathways

**Figure 3:**
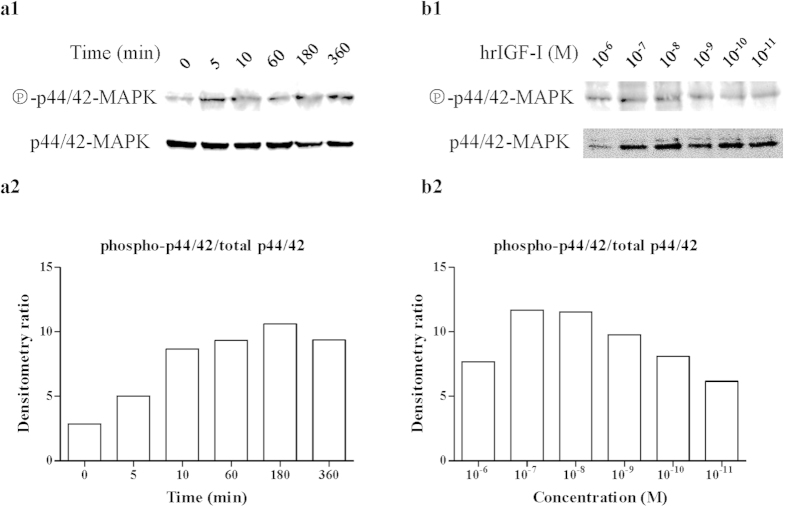
hrIGF-I stimulation leads to sustained activation of MAPK. (**a1**) Time-dependent activation of p44/42 MAPK by hrIGF-I. Oocytes were treated with hrIGF-I (10^−7^ M) for various periods of time. Lysates were analyzed by western blotting to detect the phosphorylation of p44/42 MAPK, as well as the amount of p44/42 MAPK. (**a2**) The densitometry ratio of phospho-p44/42/total p44/42 after treatment with hrIGF-I (10^−7^ M) for various periods of time. (**b1**) Dose-dependent effect of hrIGF-I on the activation of p44/42 MAPK. Oocytes were treated with hrIGF-I at various concentrations for 30 min. Lysates were subjected to western blotting with appropriate antibodies. (**b2**) The densitometry ratio of phospho-p44/42/total p44/42 after treatment with hrIGF-I at various concentrations.

To examine the effect of hrIGF-I on the activation of the MAPK and PI3K signaling cascades, *P. fucata* oocytes were treated with hrIGF-I (10^−7^ M) for various periods of time and with different concentrations of hrIGF-I for same time. The phosphorylation of p44/42 MAPK was examined by determining the ratio of phospho- to total p44/42 MAPK. As shown in Fig. 3a1, hrIGF-I-induced MAPK phosphorylation within 5 min, and the induction lasted for 6 h. The activation increased in a time-dependent manner from 0 to 180 min (Fig. 3a2). When treated for 30 min, hrIGF-I induced a dose-dependent phosphorylation of p44/42 MAPK at concentrations ranging from 10^−11^ to 10^−7 ^M (Fig. 3b1,b2).

**Figure 4:**
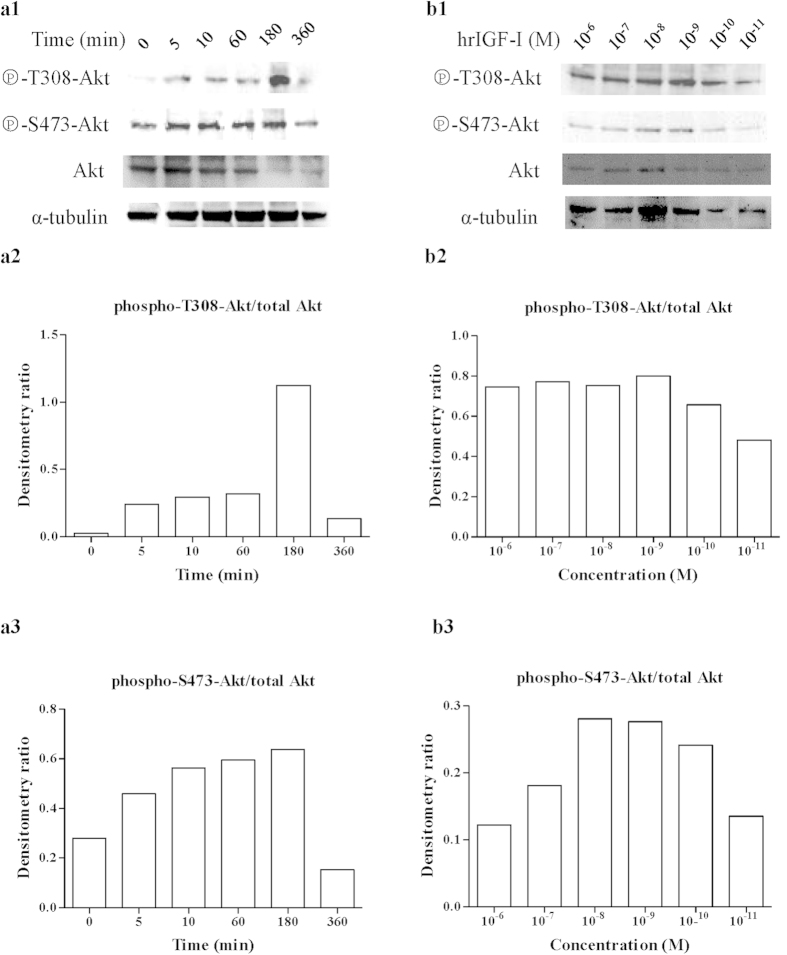
hrIGF-I stimulation leads to sustained activation of PKB/Akt. (**a1**) Time-dependent activation of PKB/Akt by hrIGF-I. Oocytes were treated with hrIGF-I (10^−7^ M) for various periods of time. Lysates were analyzed by western blotting to detect the phosphorylation of Akt/PKB at T308 and S473, as well as the amounts of Akt/PKB and α-tubulin. The gels have been run under the same experimental conditions, full-length blots of phospho-T308-Akt and α-tubulin were presented in Supplementary Figure S1. (**a2**) The densitometry ratio of phospho-T308-Akt/total Akt after treatment with hrIGF-I (10^−7^ M) for various periods of time. (**a3**) The densitometry ratio of phospho-S473-Akt/total Akt after treatment with hrIGF-I (10^−7^ M) for various periods of time. (**b1**) Dose-dependent effect of hrIGF-I on the activation of PKB/Akt. Oocytes were treated with hrIGF-I at various concentrations for 30 min. Lysates was subjected to western blotting with appropriate antibodies. (**b2**) The densitometry ratio of phospho-T308-Akt/total Akt after treatment with hrIGF-I at various concentrations. (**b3**) The densitometry ratio of phospho-S473-Akt/total Akt after treatment with hrIGF-I at various concentrations.

The results of the Akt/PKB phosphorylation indicate that hrIGF-I-induced the phosphorylation of T308 and S473 of Akt/PKB within 0 min, and the induction lasted for at least 6 h (Fig. 4a1). Phosphorylation increased in a time-dependent manner from 0 to 180 min (Fig. 4a2). The maximal activation of S473 phosphorylation occurred 180 min after hrIGF-I treatment.

This hrIGF-I-induced increase in T308 and S473 phosphorylation was dose-dependent at hrIGF-I concentrations from 10^−11^ to 10^−9^ M (Fig. 4b1). There was no significant difference in T308 phosphorylation at hrIGF-I from 10^−8^ to 10^−6^ M (Fig. 4b2). The maximal activation of S473 phosphorylation occurred at an hrIGF-I concentration of 10^−8^ M, and it decreased dose-dependently from 10^−8^ to 10^−6^ M hrIGF-I (Fig. 4b3).

### pfIRR is required for the IGF-stimulated activation of the MAPK and PI3K signaling pathways

**Figure 5:**
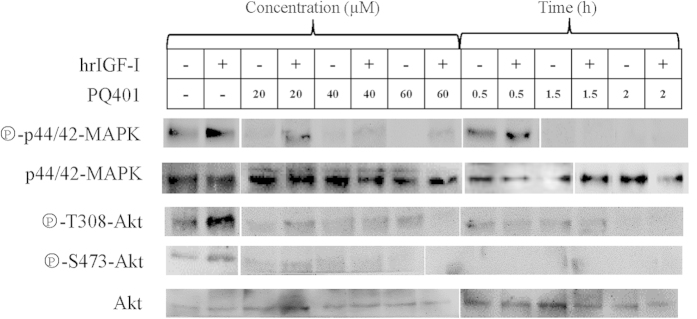
PQ401 suppresses MAPK and PKB/Akt activation. Oocytes were incubated without or with increasing concentrations of PQ401 for 1 h, or with 40 μM PQ401 for 0.5, 1.5, and 2 h, and then treated with or without hrIGF-I (10^−9^ M) for 30 min. Lysates were subjected to western blotting to detect the phosphorylation of Akt/PKB at T308 and S473, as well as the amounts of Akt/PKB, phosphorylated p44/42 MAPK, and p44/42 MAPK. “ + ” indicates treatment with hrIGF-I, and “−” indicates treatments without hrIGF-I. The gels have been run under the same experimental conditions, full-length blots of phospho-p44/42-MAPK, p44/42-MAPK and phospho-S473-Akt were presented in Supplementary Figure S2.

To assess the role of pfIRR in the hrIGF-I-mediated MAPK and PI3K signaling pathways, we measured the effects of a pfIRR inhibitor, PQ401, on hrIGF-I-mediated Akt and MAPK phosphorylation. Basal and hrIGF-I-induced phosphorylation of MAPK were lowered by a 1-h treatment with 20 μM PQ401 and by a 0.5-h treatment with 40 μM PQ401. The inhibition of MAPK phosphorylation increased in a dose-dependent manner in hrIGF-treated and untreated cells following PQ401 treatment (Fig. 5, left panel). Pre-incubation with 40 μM PQ401 for 1.5 and 2 h completely inhibited basal and hrIGF-I-stimulated MAPK phosphorylation (Fig. 5, right panel).

The basal and hrIGF-I-stimulated phosphorylation of T308 and S473 of Akt/PKB were similarly suppressed by pretreatment with PQ401. Pre-incubation with 40 μM PQ401 for 0.5 h completely prevented basal and hrIGF-I-stimulated S473 phosphorylation, whereas T308 phosphorylation was completely prevented after 2 h (Fig. 5, right panel).

### MEK is involved in hrIGF-I-mediated PI3K pathway activation

**Figure 6:**
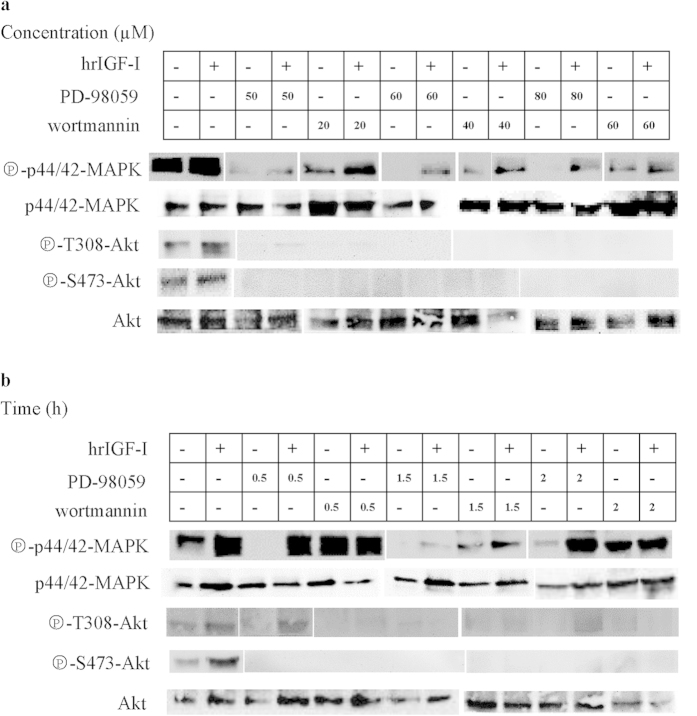
Specific inhibition of MAPK and PKB/Akt activation. (**a**) Oocytes were incubated without or with increasing concentrations of PD98059 or wortmannin for 1 h, and then treated with or without hrIGF-I (10^−9^ M) for 30 min. (**b**) Oocytes were incubated without or with 60 μM PD98059 or40 μM wortmannin for 0.5, 1.5, and 2 h, and then treated with or without hrIGF-I (10^−9^ M) for 30 min. Lysates were subjected to western blotting to detect the phosphorylation of Akt/PKB at T308 and S473, as well as the amounts of Akt/PKB, phosphorylated p44/42 MAPK, and p44/42 MAPK. “ + ” indicates treatment with hrIGF-I, and “−” indicates treatments without hrIGF-I. The gels have been run under the same experimental conditions, full-length blots of phospho-p44/42-MAPK, p44/42-MAPK, phospho-T308-Akt, phospho-S473-Akt and Akt were presented in Supplementary Figure S3 and S4.

To determine whether MEK is involved in hrIGF-I-mediated PI3K pathway activation, we assessed the effects of the MEK inhibitor PD98059 on the hrIGF-I-induced phosphorylation of T308 and S473 of Akt/PKB. Basal MAPK phosphorylation was lowered by treatment with 50 μM PD98059, and it was completely inhibited by treatment with 60 and 80 μM PD98059 for 1 h. hrIGF-I-stimulated MAPK phosphorylation was reduced by PD98059 at the three concentrations tested (50, 60, and 80 μM), and a stronger reduction was achieved by treatment with 50 μM PD98059. Pretreatment with PD98059 at 50, 60, and 80 μM concentrations for 1 h completely inhibited the basal and hrIGF-I-stimulated phosphorylation of T308 and S473 of Akt/PKB (Fig. 6a).

To detect the duration of the PD98059-meidated inhibition of PI3K activity, we analyzed the phosphorylation of T308 and S473 after treatment with 60 μM PD98059 for 0.5, 1.5, and 2 h. Basal MAPK phosphorylation was completely prevented by treatment with PD98059 for 0.5 and 1.5 h, but not for 2 h. hrIGF-I-stimulated MAPK phosphorylation exhibited the greatest decrease after 1.5 h of PD98059 treatment. Pretreatment with 60 μM PD98059 for 0.5 to 2 h completely inhibited the basal and hrIGF-I-stimulated phosphorylation of T308 and S473 of Akt/PKB (Fig. 6b).

### PI3K is involved in the hrIGF-I-mediated stimulation of the ERK pathway

To determine whether PI3K is involved in hrIGF-I-mediated ERK pathway activation, we assessed the effect of the PI3K inhibitor wortmannin on hrIGF-I-induced MAPK phosphorylation. Basal and hrIGF-I-stimulated phosphorylation of T308 and S473 were completely prevented by treatment with 20, 40, and 60 μM wortmannin for 1 h. Pretreatment with wortmannin lowered basal and hrIGF-I-stimulated MAPK phosphorylation, and a slight dose-dependent inhibition was observed (Fig. 6a).

To detect the duration of the wortmannin-mediated inhibition of ERK activity, we analyzed MAPK phosphorylation after treatment with 40 μM wortmannin for 0.5, 1, and 2 h. Basal and hrIGF-I-stimulated phosphorylation of T308 and S473 were completely prevented by treatment with wortmannin for 1.5 and 2 h. Pretreatment with 40 μM wortmannin for 1.5 h, but not for 0.5 h, resulted in the greatest decreases in basal and hrIGF-I-stimulated MAPK phosphorylation. HrIGF-I-stimulated, but not basal, MAPK activity was lowered by wortmannin treatment for 2 h (Fig. 6b).

### Effect of hrIGF-I on carbohydrate/glycogen metabolism via the PI3K and/or MAPK pathways

**Figure 7:**
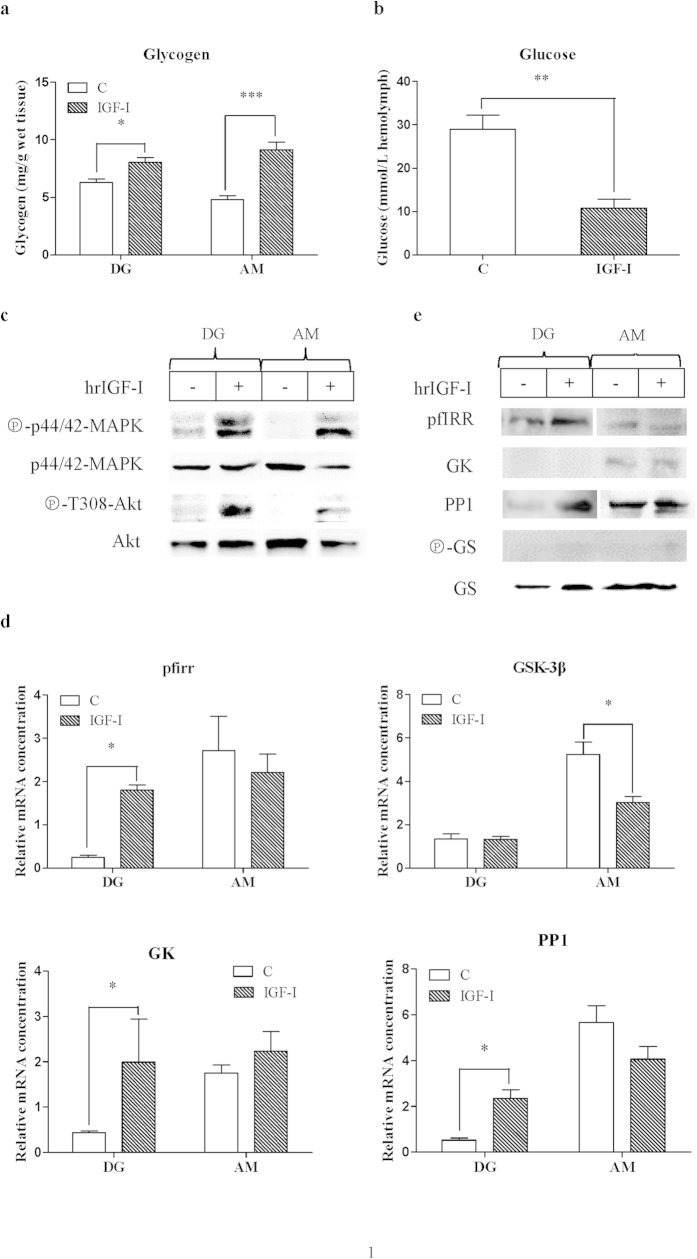
Effect of hrIGF-I on glycogen metabolism via the PI3K and/or MAPK pathways. (**a**) Changes in DG and AM glycogen contents after hrIGF-I injection. Tissue was excised from *P. fucata* injected with hrIGF-I or PBS as a control. (**b**) Glucose levels in *P. fucata* hemolymph after hrIGF-I injection. Hemolymph samples were taken from *P. fucata* injected with hrIGF-I or PBS as a control. (**c**) hrIGF-I stimulation leads to the activation of PKB/Akt and MAPK *in vivo*. Oysters were analyzed by western blotting to detect the phosphorylation of Akt/PKB at T308 and p44/42 MAPK, as well as the amounts of Akt/PKB and p44/42MAPK. (**d**) pfIRR, GK, GSK-3β, and PP1 mRNA expression in DGs and AMs of *P. fucata* after hrIGF-I injection. The mRNA levels were quantified by real-time PCR. The results are expressed as fold-changes. Each bar represents the mean ± SEM of five samples. *p < 0.05; **p < 0.01; ***p < 0.001. (**e**) Protein expression of pfIRR, GK, PP1, GS, and phospho-GS in DGs and AMs of *P. fucata* after hrIGF-I injection. The gels have been run under the same experimental conditions.

To investigate the participation and conservation of the PI3K and/or MAPK pathways in carbohydrate/glycogen metabolism in response to hrIGF-I induction, we analyzed the effects of hrIGF-I on the glycogen content and glucose levels, the phosphorylation of Akt/PKB at T308 and p44/42 MAPK, and the expression of the involved genes. Three hours after the injection of hrIGF-I or a phosphate-buffered saline (PBS) control, the glycogen content in the digestive glands (DGs) and adductor muscles (AMs) significantly increased (Fig. 7a). The glucose levels in the hemocytes of hrIGF-I-treated oysters decreased significantly compared with those of the PBS-injected control 3 h post-injection (Fig. 7b). hrIGF-I-stimulated the phosphorylation of Akt/PKB at T308, and p44/42 MAPK phosphorylation was increased compared with basal phosphorylation levels *in vivo* (Fig. 7c).

We analyzed four downstream genes of the hrIGF-I signaling pathway: the pfirr, glucokinase (GK), glycogen synthase kinase-3β (GSK-3β), and PP1 genes. The relative expression levels of the pfIRR, GK, and PP1 genes significantly increased in DGs after hrIGF-I injection, whereas there were no differences in expression levels for hrIGF-I-treated or non-treated AMs (Fig. 7d). However, GSK-3β expression decreased significantly in AMs after the injection of hrIGF-I, but it did not differ in the DGs.

The protein expression of pfIRR, GK, PP1, phospho-GS, GS, phospho-GSK-3β, and GSK-3β was measured by Western blotting. The expression of pfIRR and PP1 increased in DGs after hrIGF-I injection, whereas no difference was observed for hrIGF-I-treated or non-treated AMs (Fig. 7e), which was similar to the expression pattern of the mRNA levels. However, pfIRR expression was higher in DGs than in AMs, which differed from the pattern of mRNA expression (Fig. 7e). GK expression in the AMs was not significantly different after hrIGF-I injection, which was similar to the expression pattern of its mRNA (Fig. 7e). However, GK was not detected in the DGs, although its mRNA was expressed. GS was expressed in DGs and AMs, but phospho-GS was only detected at low levels in DGs and AMs after several hrIGF-I injections (Fig. 7e).

## Discussion

In this study, we report the generation and evaluation of a polyclonal antibody against pfIRR by expressing a fragment encoding the TK domain of pfIRR in *E. coli*. High avidity antisera were obtained, which were functional in ELISA and western blotting assays. The crude antisera that were purified by a protein A affinity column showed that the antibody is highly specific for the TK domain of pfIRR.

Via a Co-IP assay, hrIGF-I and pfIRR were shown to interact with each other. The interaction between hrIGF-I and pfIRR increased in a dose-dependent manner with the hrIGF-I concentration (10^−11^–10^−6^ M) when using the anti-hrIGF-I antibody for Co-IP (Fig. 2a); a slight dose-dependent increase was observed at hrIGF-I concentrations ranging from 10^−9^ to 10^−6^ M, and there was a dose-dependent increase at hrIGF-I concentrations from 10^−11^ to 10^−9^ M when using the anti-pfIRR antibody for Co-IP (Fig. 2b). In this study, we did not obtain the insulin protein of *P. fucata*, and in previous studies, hrIGF-I, which is structurally and functionally conserved, has been reported to be used widely in invertebrate species. Our study showed that the pfIRR gene is expressed in ovaries^39^, and a previous study showed that hrIGF-1 effects germinal cell proliferation and maturation associated with the expression of a homologous IRR in *Crassostrea gigas*^27^. Moreover, because no oyster cell lines are available presently, we used ovary cells to conduct our experiments.

Our experiments indicated that the MAPK and PI3K pathways are activated following hrIGF-I stimulation in *P. fucata* oocytes, although their threshold concentrations and durations differed. The effect of hrIGF-I on MAPK activation was sustained for at least 6 h, and the maximal activation required a relatively high concentration of hrIGF-I (10^−7^ M). The effect of hrIGF-I on Akt activation was also sustained for at least 6 h. The maximal activation of T308 phosphorylation was obtained at 10^−9^ M hrIGF-I, while the maximal activation of S473 phosphorylation occurred at 10^−8^ M hrIGF-I. Although the maximal activation level of phospho-T308-Akt was achieved at 180 min, the signal was very strong compared with that in the other lanes; thus, it may be an outlier.

Full Akt activation requires the dual phosphorylation of S473 by mammalian target of rapamycin companion 2 (mTORC2)–rapamycin-insensitive companion of mTOR complex (RICTOR), integrin-linked kinase 1 (ILK)-1, or DNA-dependent protein kinase (DNAPK) as well as the phosphorylation of T308 by PDK1^40^. PDK1 is known to function downstream of PI3K. PI3K activation results in the production of phosphatidylinositol 3,4,5 trisphosphate (PIP3), which binds to the pleckstrin homology (PH) domain of PDK1 and thereby recruits PDK1 to the plasma membrane^41^. Thus, we examined T308 and S473 phosphorylation of Akt, and hrIGF-I resulted in a stronger activation of S473 than T308. As is known, the recruitment of Akt to the plasma membrane is required for the phosphorylation of S473, and PI3K activity is thought to be necessary for S473 phosphorylation^42^,^43^.

In previous studies, a diaryl urea compound, PQ401, was found to antagonize IGF-IR autophosphorylation in cultured human MCF-7 cells, with a half-maximal inhibitory concentration of 12 μM, as well as the growth of cultured breast cancer cells in serum at 10 μM^44^. Because the autophosphorylation regions of pfIRR and IGF-IR are highly conserved, we used PQ401 to inhibit pfIRR. Our previous studies have indicated that PQ401 decreases pfIRR mRNA expression and leads to the developmental arrest of *P. fucata* embryos^39^. In this present study, inhibition of pfIRR by PQ401 decreased the activation of MAPK and Akt, which indicated that pfIRR is required for the basal and IGF-mediated activation of the MAPK and PI3K signaling pathways. The results suggest that treatment with 40 μM PQ401 for 1.5 h may be the most efficient conditions for inhibiting MAPK activation, and treatment with 40 μM PQ401 for 2 h may be the most efficient conditions for inhibiting Akt activation among the different doses of PQ401 and different treatment durations used in our experiments.

We used the MEK inhibitor PD98059 and the PI3K inhibitor wortmannin to examine the role of MAPK and PI3K in regulating the hrIGF-I signaling pathway. Treatments with PD98059 inhibited basal and hrIGF-I-mediated MAPK and Akt activities, indicating that hrIGF-I activates the MAPK signaling pathway, and that MEK is involved in hrIGF-I-mediated PI3K pathway activation. In mammals, active ERK can influence the PI3K/Akt pathway via phosphorylation of GAB1 and IRS on several serine residues that are adjacent to p85 PI3K-binding sites (three YXXM motifs)^45^. The GAB1–p85 PI3K complexes alleviate the intrinsic inhibition of PI3K, which further increases PIP3 production. PIP3 in the plasma membrane leads to the membrane recruitment of GAB1 and IRS through their PH domains, thereby generating a positive feedback loop^46^,^47^. In Ras/MAPK → PI3K/Akt cross-talk, in some cells, ERK inhibition by U0126 resulted in a decrease in Akt activation after hepatocyte growth factor (HGF) stimulation^48^. However, this positive regulatory effect of Ras/MAPK on PI3K depends on the type of stimulation, as it has the opposite effect on epidermal growth factor (EGF) signaling. The Ras/MAPK cascade negatively regulates PI3K after EGF stimulation^49^,^50^. In *P. fucata*, the results of hrIGF-I signaling show that PI3K activity is decreased following MAPK inhibition, as also occurs during HGF signaling.

PD98059 inhibited MAPK activation at 50–80 μM concentrations. T The reduced but not inhibited MAPK activation by hrIGF-I stimulated demonstrated that MAPK activation is not regulated by only one signaling pathway, and it indicates that Ras/MAPK → PI3K/Akt cross-talk that can amplify MAPK signaling occurs upstream of MEK. The maximal inhibition time for PD98059 in our study was 2 h. The positive influence of the Ras/MAPK pathway on the PI3K/Akt pathway was not affected by the inhibitor concentration or the stimulation time, which may be because the signal strength (hrIGF-I dose) and the stimulation time (0.5 h) in these inhibition experiments were the same. The basal activities of MAPK and Akt were inhibited by different doses of PD98059 and different treatment times, whereas after treatment, hrIGF-I stimulated the phosphorylation of MAPK, but not Akt, at either T308 or S473. This may be because MAPK is more sensitive than Akt to the dose of hrIGF-I used (10^−9^ M).

Treatments with the PI3K inhibitor wortmannin reduced basal and hrIGF-I-mediated Akt and MAPK activities, indicating that hrIGF-I activates the MAPK signaling pathway, and that PI3K is involved in the hrIGF-I-mediated induction of the MAPK pathway. Inhibition of Akt activity by wortmannin indicates that it functions by inhibiting the PI3K–AKT pathway in *P. fucata*. Inhibition of PI3K by wortmannin and LY294002 has been reported to block the activation of ERK1/2 in the rat skeletal muscle cell line L6^51^, Chinese hamster ovary cells^52^, and rat adipocytes^53^, but not in other cell types such as rabbit parietal cells^54^,^55^. Previous studies have shown that PI3K positively regulates the Ras/MAPK cascade in most cellular systems, which was also shown in our study. In mammals, PI3K affects MAPK signaling primarily at the level of PIP3-mediated Ras guanine nucleotide exchange factor and Ras GTPase activating protein (GAP) signaling. The positive PI3K–PIP3–GAB–PI3K feedback loop leads to the phosphorylation of GAB1 and IRS, which can recruit Grb2 and Son of Sevenless, leading to Ras activation^56^,^57^,^58^ or bind RasGAP, which catalyzes Ras deactivation^59^. GAB1 association with SHP2 increases SHP2 phosphatase activity by dephosphorylating SHP2 substrates, such as RasGAP and c-Src. The resulting activation of c-Src and Ras leads to an up-regulation of ERK signaling^60^,^61^.

Wortmannin inhibited Akt activation at 20–60 μM concentrations. A previous study demonstrated that Ras/MAPK → PI3K/Akt cross-talk is dependent on the concentrations of growth factors. For example, at saturating EGF doses, PI3K inhibition by wortmannin only slightly attenuates ERK phosphorylation in A431 human epidermoid carcinoma and MCF7 human mammary carcinoma cells (over the 2–60 min response period), whereas PI3K inhibition dramatically decreases ERK phosphorylation at low physiological EGF doses^13^. Wortmannin decreased MAPK phosphorylation in the middle and late time points of signal propagation, but the peak of activation (0.5 h) did not differ from that of control cells. This decreasing sensitivity of MAPK phosphorylation to PI3K inhibition was also observed in human embryonic kidney 293, HeLa, T47D, and BT-474 cells^13^. A 10^−9^ M concentration of hrIGF-I dramatically decreased MAPK phosphorylation with increasing wortmannin concentration, but it had a smaller effect on the inhibition time, which indicates that 1 nM hrIGF-I may be a modest dose.

Our experiments on MAPK and PI3K signaling in *P. fucata* in regard to carbohydrate/glycogen metabolism induction by hrIGF-I demonstrated that the mechanism of the hrIGF-I responsive machinery is conserved in *P. fucata*. The glycogen content in various tissues (the mantle, gill, AMs, foot, gonad, and DGs) was examined (Supplementary Figure S5). The glycogen content ranged from 1.69 to 4.4 mg/g wet mass, and was highly detectable in DGs and AMs, and at low levels in the mantle, which is similar to a previous study in *Crassostrea angulate*^62^. Although muscle is not a glycogen storage tissue in oysters, because its level does not exceed 5% of the total biochemical content^63^, muscle is a repository of active glycogenolysis that quickly provides ATP for muscular contraction in many species^64^. In oysters, most tissues, such as in AMs, DGs, and gills, are capable of glycogen hydrolysis and/or glucose formation^63^,^65^. Therefore, we determined the glycogen contents after hrIGF-I injection in DGs and AMs.

Our data obtained for DGs and AMs of hrIGF-I-treated oysters showed higher glycogen contents 3 h post-injection compared with those of controls, and lower glucose levels in hemolymph than in the controls. This indicates that the increase in glycogen contents in DGs and AMs is correlated with the decreased glucose levels in the hemolymph. Similarly, in the white shrimp *P. vannamei*, bovine insulin has an effect on glucose uptake in the hepatopancreas^37^. Meanwhile, hrIGF-I stimulation increases the phosphorylation of Akt/PKB at T308 and p44/42 MAPK, which indicates that the PI3K and MAPK pathways may participate in regulating the effect of hrIGF-I on carbohydrate/glycogen metabolism in *P. fucata.* As described above, this may be caused by the cross-talk between PI3K and MAPK, although the mechanism is still unclear and needs to clarify in the future.

The mRNA expression of the four examined genes was higher in AMs than in DGs, although the expression of three of the mRNAs increased in DGs after the injection of hrIGF-I. The mRNA and protein expression of pfIRR increased in DGs after injection, whereas no difference was observed in AMs. pfIRR may have a transient effect in AMs, and long-term regulatory effects in DGs. The three downstream genes of the hrIGF-I signaling pathway were chosen based on the results of an RNA sequencing analysis in our previous study^66^. As is known, insulin inactivates GSK-3β by Akt-mediated phosphorylation of Ser9, reducing the GSK3β inhibitory activity toward GS to facilitate glycogen synthesis^67^. Our data for AMs is exactly the same as in previous reports, and it indicates that the function of GSK-3β is conserved in *P. fucata.* However, the expression of GSK-3β was not changed in DGs. This may be because GSK3 expression may have changed before or after the 3-h post-injection time of hrIGF-I. A study of the white shrimp *P. vannamei* has shown that the glycogen content fluctuates in DGs after hrIGF-I injection: the glycogen content in the hepatopancreas fell significantly within 1 h post-injection of hrIGF-I, increased significantly 3 h post-injection, and decreased significantly 5 h post-injection^37^. GS is expressed in DGs and AMs, but phospho-GS was only detected at low levels in DGs and AMs after hrIGF-I injection, as determined by western blotting. This may be caused by the species diversity of the antibody. However, the low level of phospho-GS still proved that the function of GS in glycogen synthesis is conserved in *P. fucata*.

GK is an enzyme that facilitates the phosphorylation of glucose to glucose-6-phosphate, and it plays an important role in the regulation of carbohydrate metabolism by acting as a glucose sensor, triggering shifts in metabolism in response to rising or falling levels of glucose^68^. PP1 plays a crucial role in the regulation of blood-glucose levels in the liver, as well as glycogen metabolism^35^. The mRNA expression of the GK and PP1 genes increased significantly in DGs, suggesting that their function in regulating glycogen content is conserved. The expression of PP1 protein was consistent with its mRNA expression pattern, but GK was not detected in DGs, which may be caused by the different responses of antibodies in different tissues, and the species diversity of the antibody. However, GK mRNA and protein expression did not change in AMs after the injection of hrIGF-I. We speculate that GK and PP1 may have transient effects in AMs, and long-term regulatory effects in DGs. In mammals, GK is expressed mostly in the liver, pancreas, small intestine, and brain, where it plays crucial roles in responding to rising or falling levels of blood glucose^69^. The functions of GK and PP1 in AMs need to be investigated in the future.

In conclusion, the present study shows that pfIRR interacts with hrIGF-I, and stimulates the MAPK and PI3K signaling pathways in *P. fucata* oocytes, and that there is cross-talk between the MAPK and PI3K/Akt pathways, in which MEK positively regulates the PI3K pathway and PI3K positively regulates the MAPK cascade. Additionally, the i.m. injection of hrIGF-I stimulates the PI3K and MAPK pathways to increase glucose transport, glycogen synthesis, and it also regulates the expression of specific genes. In the future, the multiple signaling nodes and routes involved in cross-talk between these two pathways, as well as the functions of MAPK and PI3K signaling in *P. fucata* in other physiological or environmental contexts, should be more thoroughly investigated.

## Methods

### Animals and chemicals

*P. fucata* (2 years old) were obtained from the Marine Biology Research Station at Daya Bay of the Chinese Academy of Sciences (Shenzhen, Guangdong, China). The oysters were cultivated in floating net cages in the sea under natural conditions. All animal experiments were conducted in accordance with the guidelines and approval of the respective Animal Research and Ethics Committees of the Chinese Academy of Sciences.

Oocytes were obtained directly from the gonad with a Pasteur pipet, filtered through 120-μm nylon mesh to remove debris, and washed three times with filtered (0.45-μm filter) natural seawater at room temperature (26 °C).

Reagents were obtained from the following sources: hrIGF-I from Shanghai Prime Gene Bio-Tech. Co., Ltd. (Shanghai, China); dimethyl sulfoxide from Sigma–Aldrich (St. Louis, MO, USA); hrIGF-I monoclonal antibody from Abcam (San Francisco, CA, USA); rabbit IgG from Beyotime (Jiangsu, China), phospho-T308 and phospho-S473Akt/PKB, Akt/PKB, phospho-p44/p42 MAPK/Erk 1/2, p44/p42 MAPK/Erk 1/2 antibodies, wortmannin, and PD98059 from Cell Signaling Technology (Danvers, MA, USA); GK antibody, GS antibody and PP1 (catalytic subunit, alpha isozyme) antibody from Sangon Biotech (Shanghai, China); phospho-GS (Ser641) antibody from Sino Biological (Beijing, China); alpha-tubulin polyclonal antibody from Proteintech (Chicago, IL, USA); horseradish peroxidase (HRP)-conjugated goat anti-rabbit IgG secondary antibody from Abbkine (Redlands, CA, USA).

### Antibody production

A polyclonal antibody against *P. fucata* IRR was made by Shanghai Biomodel Organism Science & Technology Development Co., Ltd (Shanghai, China). An 837-bp cDNA fragment encoding the TK domain of IRR (GenBank accession number: JX121113) was amplified and cloned into the BamHI/XhoI sites of the pET28a expression vector (TAKARA, Japan), and histidine-tagged TK was expressed in *E. coli* and purified with a Ni^2+^-NTA column.

Polyclonal antibody was raised against the purified TK domain in two rabbits. After immunization, the antisera were collected and purified by a protein A affinity column. The titers of the antisera were determined by ELISA. Western blot analyses were conducted to characterize the polyclonal antibody.

### Co-IP

Oocytes were treated with different concentrations of hrIGF-I for 4 h at 4 °C. hrIGF-I was dissolved in PBS (0.2 M) at a concentration of 0.02 mM, and then the oocytes were separately treated with 10^−6^, 10^−7^, 10^−8^, 10^−9^, 10^−10^, and 10^−11^ M hrIGF-I. The oocytes were lysed and immunoprecipitated using the Pierce Classic IP Kit (#26146; Pierce, Rockford, IL, USA). The lysates were co-immunoprecipitated using anti-hrIGF-I (10 μg) antibodies, subjected to SDS-PAGE (10%), and detected by western blotting with an anti-pfIRR antibody (10 μg). Rabbit IgG served as a negative control. The Co-IP lysates obtained using anti-pfIRR antibodies (15 μg) were detected by western blotting with anti-hrIGF-I (10 μg). IgG served as a negative control.

### Western-blot assays

Cell lysates were separated by SDS-PAGE. After transfer to polyvinylidene fluoride membranes (0.22-mm pore size, EMD Millipore, Billerica, MA, USA), the membranes were blocked in 5% bovine serum albumen (BSA) (Thermo Fisher Scientific) in Tris-buffered saline-Tween 20 (TBST). The blots were incubated with 1:1,000 to 1:5,000 dilutions of the indicated antibodies in blocking buffer overnight at 4 °C. Then, blots were washed with TBST and incubated with a 1:5,000 dilution of an HRP-conjugated anti-rabbit secondary antibody in blocking buffer for 1 h at room temperature, followed by further washing. The SuperSignal® West Pico chemiluminescent substrate was used to detect HRP according to the manufacturer’s instructions (Pierce). Positive signals from the target proteins were visualized using a TANON5200 image analyzer (Bio-tanon, Shanghai, China).

### hrIGF-I stimulation *in vitro*

Oocytes were incubated with hrIGF-I (10^−7^ M) for various periods of time (0, 5, 10, 60, 180, and 360 min), and then analyzed by western blotting to detect the phosphorylation of Akt/PKB at T308 and S473, as well as the amounts of Akt/PKB, phosphorylated p44/42 MAPK, p44/42 MAPK, and α-tubulin. Oocytes were incubated with hrIGF-I at various concentrations (10^−6^, 10^−7^, 10^−8^, 10^−9^, 10^−10^, and 10^−11^ M) for 30 min. Lysates was subjected to western blotting with appropriate antibodies. The densitometry ratio of phospho-p44/42/total p44/42, and phospho-Akt/total Akt were measured by Tanon Gel Imaging System software.

### pfIRR inhibition *in vitro*

Oocytes were incubated without or with increasing concentrations of PQ401 for 1 h, or with 40 μM PQ401 for 0.5, 1.5, and 2 h, and then treated with or without hrIGF-I (10^−9^ M) for 30 min. Lysates were subjected to western blotting to detect the phosphorylation of Akt/PKB at T308 and S473, as well as the amounts of Akt/PKB, phosphorylated p44/42 MAPK, and p44/42 MAPK.

### MEK inhibition and Akt/PKB inhibition *in vitro*

Oocytes were incubated without or with increasing concentrations of PD98059 or wortmannin for 1 h, or with 60 μM PD98059 or 40 μM wortmannin for 0.5, 1.5, and 2 h, and then treated with or without hrIGF-I (10^−9^ M) for 30 min. Lysates were subjected to western blotting to detect the phosphorylation of Akt/PKB at T308 and S473, as well as the amounts of Akt/PKB, phosphorylated p44/42 MAPK, and p44/42 MAPK.

### hrIGF-I stimulation *in vivo*

Oysters were injected i.m. with hrIGF-I (0.06 μg/g wet body weight) or an equal volume of PBS as a control for 3 h. The oysters were removed for hemolymph sampling and then sacrificed. Hemolymph samples were processed immediately. Glucose levels were determined according to the instructions of the manufacturer of the Glucose Assay Kit (Nanjing Jiancheng Bioengineering Institute, China). The DGs and AMs were quickly dissected and divided into three parts. One part was processed immediately for glycogen content according to the instructions of the manufacturer of the Liver/Muscle Glycogen Assay Kit (Nanjing Jiancheng Bioengineering Institute), while another was frozen in liquid nitrogen and stored at −80 °C for subsequent RNA extraction. Real-time polymerase chain reaction (PCR) was performed as described previously^39^. Primers for four genes involved in the hrIGF-I signaling pathway, the pfIRR, GK, GSK-3β, and PP1 genes, as well as 18 S RNA and β-actin genes, are shown in Table 2. The third portion was subjected to western blotting to detect the phosphorylation of Akt/PKB at T308, as well as the amounts of Akt/PKB, phosphorylated p44/42 MAPK, p44/42 MAPK, pfIRR, GK, PP1, GS, and phospho-GS.

## Additional Information

**How to cite this article**: Shi, Y. and He, M.-x. PfIRR Interacts with HrIGF-I and Activates the MAP-kinase and PI3-kinase Signaling Pathways to Regulate Glycogen Metabolism in *Pinctada fucata*. *Sci. Rep.* **6**, 22063; doi: 10.1038/srep22063 (2016).

## Supplementary Material

Supplementary Information

## Figures and Tables

**Table 1:** Anti-TK serum titers were tested by ELISA; dilution ranged from 1:1,250 to 1:80,000.

	1250	2500	5000	10,000	20,000	40,000	80,000	Rabbit pre-immune serum (1:1,250)
Antiserum	2.375	2.376	2.375	2.359	1.817	0.942	0.497	0.072

Rabbit pre-immune serum was used as a negative control.

**Table 2:** Primer sequences for the amplification of target genes selected from an RNA sequencing study and GenBank.

Annotation	Sequence name	Primer name	Primer sequence (5′ to 3′)
Glucokinase	Unigene101416	101416 F	ATTCCGACTCCCTTGGTATG
		101416 R	GACGGGTGATTTGTCTTTTT
Glycogen synthase kinase-3β	Unigene102110	102110 F	CAACCACCCGACTTCCTAAC
		102110 R	GGATGCCCTTCATTCCCAGC
Protein phosphatase 1	Unigene102714	102714 F	TGGTGAAGACAAAAGTGGAT
		102714 R	TGGTCATATGTTGAGAGGTG
Pfirr	JX121113	irrF	AGACGGAGACGGGAAAGAAG
		irrR	CCCCCAACAGACGTACAACA
18 S RNA	AY877529	18sF	CGTTTCAACAAGACGCCAGTAG
		18sR	ACGAAAAAAAGGTTTGAGAGACG
β-actin	AB252571	β-actinF	TGGTATGGGACAGAAGGAC
		β-actinR	GACAATGCCGTGCTCAAT
