# Supplementary material for: PfIRR Interacts with HrIGF-I and Activates the MAP-kinase and PI3-kinase Signaling Pathways to Regulate Glycogen Metabolism in Pinctada fucata
Source: Sci Rep. 2016 Feb 25;6:22063. doi: 10.1038/srep22063 (PMC4766514; doi:10.1038/srep22063)
Supplement: Supplementary Information [file srep22063-s1.doc]

**PfIRR Interacts with** **HrIGF-I and Activates the MAP-kinase and PI3-kinase Signaling Pathways to Regulate Glycogen Metabolism in** ***Pinctada fucata***

**Yu Shi^1^ and** **Mao-xian He^1^***

^1^CAS Key Laboratory of Tropical Marine Bio-resources and Ecology, Guangdong Provincial Key Laboratory of Applied Marine Biology, South China Sea Institute of Oceanology, Chinese Academy of Sciences, 164 West Xingang Road, Guangzhou 510301, China

*corresponding. [hmx@scsio.ac.cn](mailto:hmx@scsio.ac.cn)


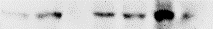
**a**


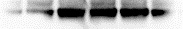
**b1**


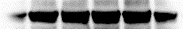


**b2**

**Supplementary Fig. S1.** Full-length blots of phospho-T308-Akt and α-tubulin presented in Figure 4a. **a.** Full-length blots of phospho-T308-Akt in lane 1-6. **b1 and b2.** Full-length blots of α-tubulin in lane 1 and lane 2-5.


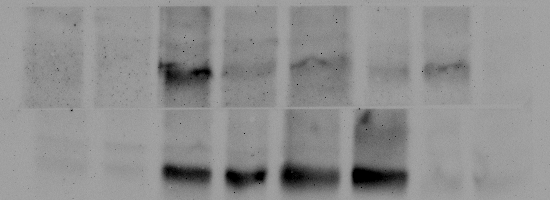
**a**

**
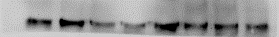
**

**b1**

**
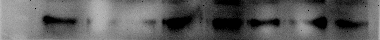
**

**b2**


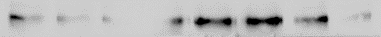
**b3**


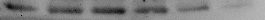
**c**

**Supplementary Fig. S2.** Full-length blots of phospho-p44/42-MAPK, p44/42-MAPK and phospho-S473-Akt presented in Figure 5. **a.** Full-length blots of phospho-p44/42-MAPK in lane 1-14. **b1, b2 and b3.** Full-length blots of p44/42-MAPK in lane 1-2 and lane 9-11 and lane 12-14. **c**. Full-length blots of phospho-S473-Akt in lane 1-2.


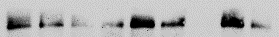
**a1**


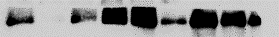
**a2**

**
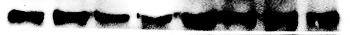
b**

**
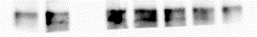
**

**c**

**
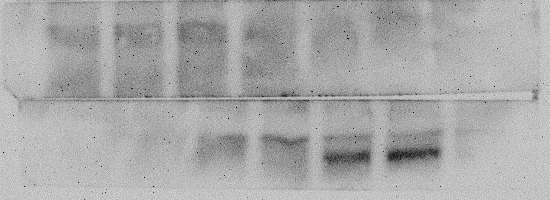
**

**d**


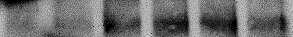
**e1**


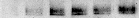
**e2**

**Supplementary Fig. S3.** Full-length blots of phospho-p44/42-MAPK, p44/42-MAPK, phospho-T308-Akt, phospho-S473-Akt and Akt presented in Figure 6a. **a1 and a2.** Full-length blots of phospho-p44/42-MAPK in lane 1-14. **b.** Full-length blots of p44/42-MAPK in lane 9-14. **c.** Full-length blots of phospho-T308-Akt in lane 1 and 2. **d**. Full-length blots of phospho-S473-Akt in lane 1-14. **e1 and e2**. Full-length blots of Akt in lane 1-4 and lane 11-14.


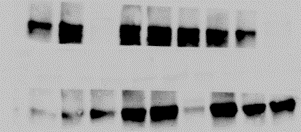
**a**


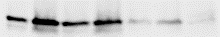
**b1**


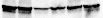
**b2**


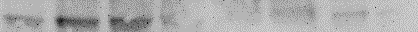
**c**


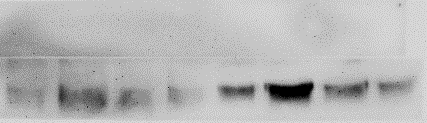
**d**

**Supplementary Fig. S4.** Full-length blots of phospho-p44/42-MAPK, p44/42-MAPK, phospho-T308-Akt and phospho-S473-Akt presented in Figure 6b. **a.** Full-length blots of phospho-p44/42-MAPK in lane 1-14. **b1 and b2.** Full-length blots of p44/42-MAPK in lane 7-10 and lane 11-14. **c.** Full-length blots of phospho-T308-Akt in lane 1-8. **d**. Full-length blots of phospho-S473-Akt in lane 1-8.

**Supplementary Fig. S5.** Changes of glycogen contents in various tissues of *P. fucata*. M, mantle; G, Gill; AM, adductor muscle; F, Foot; GD, gonad; DG, digestive gland. Each value represents means ± SEM of 5 assays.
